# Long-term intake of *Lactobacillus paracasei* KW3110 prevents age-related chronic inflammation and retinal cell loss in physiologically aged mice

**DOI:** 10.18632/aging.101583

**Published:** 2018-10-19

**Authors:** Yuji Morita, Kenta Jounai, Akihiko Sakamoto, Yasuyuki Tomita, Yoshihiko Sugihara, Hiroaki Suzuki, Konomi Ohshio, Masato Otake, Daisuke Fujiwara, Osamu Kanauchi, Mitsuo Maruyama

**Affiliations:** 1 Research Laboratories for Health Science & Food Technologies, Kirin Company, Ltd., Yokohama, Kanagawa, Japan; 2 Technical Development Center, Koiwai Dairy Products Co Ltd., Sayama, Saitama 350-1321, Japan; 3 Department of Mechanism of Aging, National Center for Geriatrics and Gerontology, Obu, Aichi 474-8511, Japan; 4 Department of Aging Research, Nagoya University Graduate School of Medicine, Nagoya, Aichi 466-8550, Japan

**Keywords:** *Lactobacillus paracasei* KW3110, age-related inflammation, proinflammatory cytokine, retina

## Abstract

Age-related chronic inflammation is a major risk factor for the incidence and prevalence of age-related diseases, including infectious and neurodegenerative diseases. We previously reported that a lactic acid bacteria, *Lactobacillus paracasei* KW3110, activated macrophages and suppressed inflammation in mice and humans. In this study, we investigated whether long-term intake of heat-killed L. paracasei KW3110 modulated age-related inflammation and altered the gut microbiota in physiologically aged mice. Compared with age-matched control mice, fecal analyses of gut microbiota revealed that intake of *L. paracasei* KW3110 mitigated age-related changes of beneficial bacterial composition, including the *Bifidobacteriaceae* family. *L. paracasei* KW3110 intake also mitigated age-related immune defects by reducing the prevalence of interferon-gamma (IFN-γ) -producing inflammatory CD4-positive T cells in the lamina propia of the small intestine, and reduced serum levels of proinflammatory cytokines. Furthermore, *L. paracasei* KW3110 intake suppressed retinal inflammation by reducing proinflammatory cytokine-producing macrophage, and age-related retinal cell loss. Taken together, these findings suggested that *L. paracasei* KW3110 mitigated age-related chronic inflammation through modulation of gut microbiota composition and immune system functions in aged mice, and also reduced age-related retinal ganglion cell (RGC) loss. Further studies are needed to evaluate the effect in age-related senescent changes of the retina.

## Introduction

Aging involves a progressive decline of physiological functions in various organs, influenced by several factors, including genetic factors and environmental factors [1–3]. As the aged population has been growing rapidly around the world, the therapeutic and preventive approaches to decelerate senescence are of great concern. Among the features of aging, the decline in immune function has been widely examined, because it results in chronic low grade inflammation, which is a major risk factor for the incidence and prevalence of age-related diseases, including infectious diseases, tumors, and neurodegenerative diseases [4–8].

The retina, one of the neural tissues, is also affected by chronic low grade inflammation. Age-related retinal neurodegenerative diseases, such as age-related macular degeneration (AMD), are major causes of blindness in the elderly [9–12]. The disease is caused by age-related retinal cell loss, including retinal ganglion cell (RGC) death [13] and photoreceptor cell death [14,15], at least partly due to chronic inflammation [16–19]. Several therapeutic pharmacological agents for suppression of retinal diseases have been reported [20,21]. However, human eyes are exposed to daily chronic stress, such as photo-oxidative stress, and as a result, safe and long-term approaches based on diet to mitigate retinal chronic inflammation are especially attractive.

Age-related immune dysfunctions leading to chronic inflammation have been previously reported. Thymic involution and disruption of homeostatic T cell proliferation, including decreased numbers of naïve T cells, accumulation of memory T cells, and increased numbers of regulatory T cells (Tregs), have been studied [22–24], and altered numbers of B cells with aging, reduced antibody production, and age-related dysfunction of other innate immune cells have also been reported [25–30]. Although some food materials or constituents, for example, prebiotics and probiotics, can improve age-related immune defects [31–34], their mechanism remains poorly understood. Recent studies suggested that the gut microbiota composition may be associated with age-related immune dysfunctions [35–37]. Disruption of gut microbiota composition has been also implicated in retinal diseases, including AMD, through a gut-retina axis [38]. Therefore, preventive dietary approaches involving alterations of gut microbiota composition for improving age-related retinal chronic inflammation should be studied.

Lactic acid bacteria are widely consumed as probiotics and paraprobiotics to enhance gut barrier function and improve immune systems. Studies have also demonstrated functional roles of several lactic acid bacterial strains in humans, including for the prevention of diarrhea, allergies, and metabolic disorders [39]. However, the long-term effects of lactic acid bacteria on age-related chronic inflammation remain unclear. We previously reported that *Lactobacillus paracasei* KW3110 activated macrophages and suppressed excessive inflammation in mice and humans [40–43]. In this study, we demonstrated the suppressive effects of the long-term intake of *L. paracasei* KW3110 on age-related alterations of gut microbiota composition and expansion of inflammatory CD4-positive T cells in the lamina propria of the small intestine (SI-LP). Furthermore, we also revealed the protective effects of the long-term intake of *L. paracasei* KW3110 on age-related retinal cell loss. We proposed that the long-term intake of *L. paracasei* KW3110 contributed to the prevention of chronic inflammation and age-related retinal cell loss in physiologically aged mice.

## RESULTS

### Intake of *L. paracasei* KW3110 affected bacterial flora in aged mice

The gut microbiota plays a critical role in the immune system, and aging has been reported to alter gut bacterial flora composition [35]. Previous studies have reported that some prebiotics and probiotics can alter gut bacterial flora composition and improve immune defects [44,45]. Therefore, to investigate whether intake of *L. paracasei* KW3110 affected the gut microbiota composition in aged mice, 16-month-old mice were fed a diet with or without *L. paracasei* KW3110 for 6 months. We analyzed bacterial 16S ribosomal RNA gene sequences in the feces. The microbiota composition at the phylum level revealed that the *Firmicutes*/*Bacteroidetes* ratio was lower in aged mice fed a control diet than in young mice fed a control diet (Fig. 1A, B). This result is consistent with a previous report [46]. However, in aged mice fed a diet containing *L. paracasei* KW3110 for 6 months, the *Firmicutes*/*Bacteroidetes* ratio was decreased compared with that in age-matched control mice (Fig. 1B).

**Figure 1AB:**
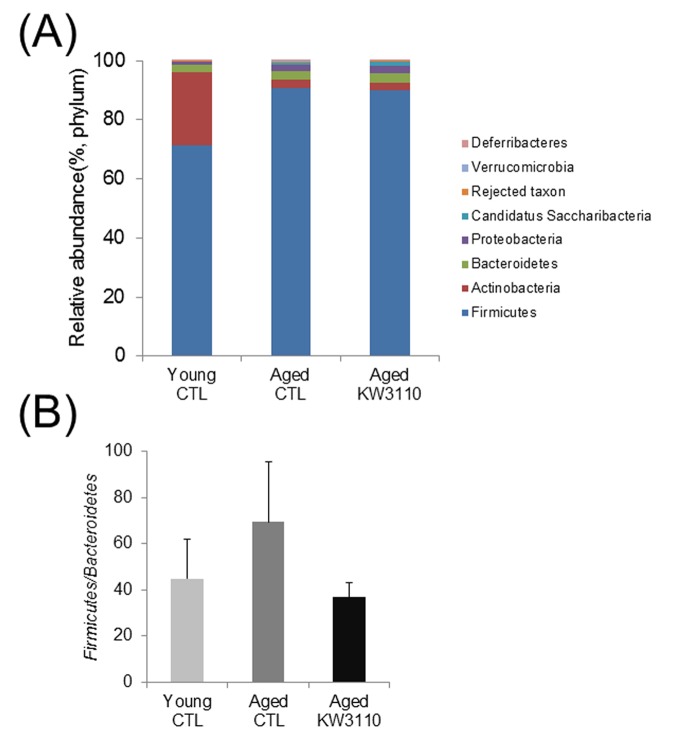
**The intake of *Lactobacillus paracasei* KW3110 in aged mice affected the gut microbial composition.** Feces were collected and subjected to flora analysis in young (3-months-old) and aged mice (22-months-old). (**A**) Distribution of gut microbiota (% of total 16S rDNA) at the phylum level. (**B**) Comparison of the *Firmicutes* to *Bacteroidetes* ratio. Values are presented as the means ± SEM of relative abundance of each phylum.

At the bacterial family level, the bacterial ratios in the feces were altered in aged mice as compared with young control mice (Fig. 1C). In the aged mice groups, the intake of *L.* *paracasei* KW3110 affected some bacterial abundances. For example, the mean relative abundances of *Peptostreptococcaceae* (*p* = 0.011) and *Bifidobacteriaceae* (*p* = 0.038) were significantly higher in aged mice fed a diet containing *L. paracasei* KW3110 for 6 months than in age-matched mice fed a control diet (Fig. 1D). In contrast, the mean relative abundance of *Streptococcaceae* was significantly lower (*p* = 0.0079) in aged mice fed a diet containing *L. paracasei* KW3110 than in age-matched mice fed a control diet (Fig. 1D).

**Figure 1CD:**
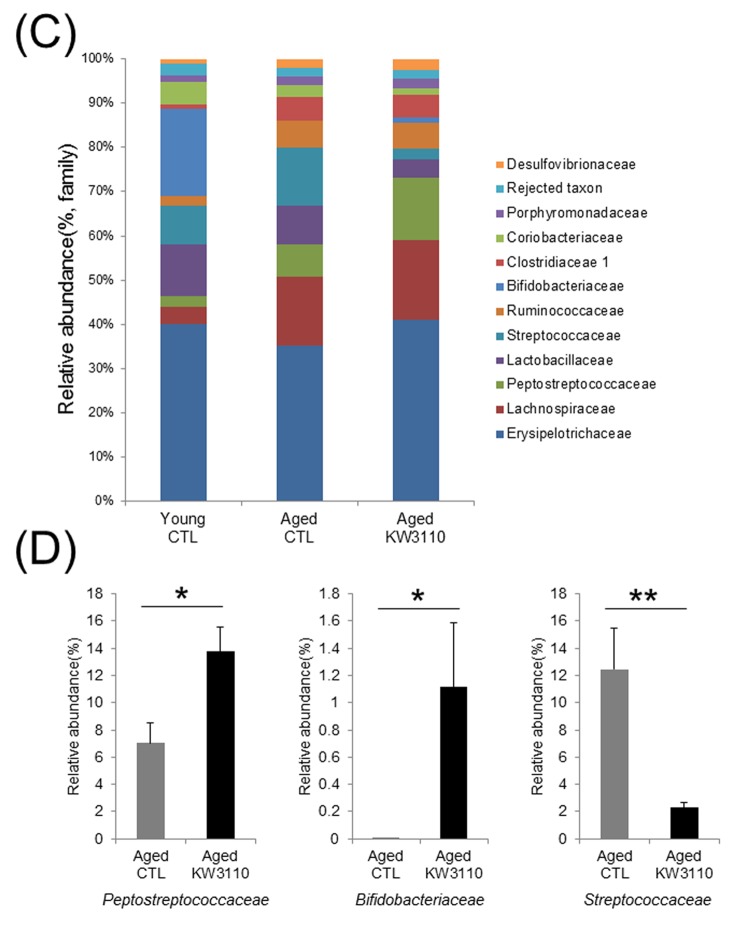
**The intake of *Lactobacillus paracasei* KW3110 in aged mice affected the gut microbial composition.** (**C**) Distribution of gut microbiota (% of total 16S rDNA) at the family level. Families with proportions less than 1% are not listed. (**D**) Comparisons of relative abundances of *Peptostreptococcaceae* (left panel), *Bifidobacteriaceae* (middle panel), and *Streptococcaceae* (right panel) families. Values are presented as the means ± SEM. Significance was assumed if the p value was < 0.05. ^*^p < 0.05, ^**^p < 0.01.CTL = control diet; KW3110 = *Lactobacillus paracasei* KW3110 diet

### The intake of *L. paracasei* KW3110 affected the lymphocyte subpopulation of SI-LP in aged mice

**Figure 2:**
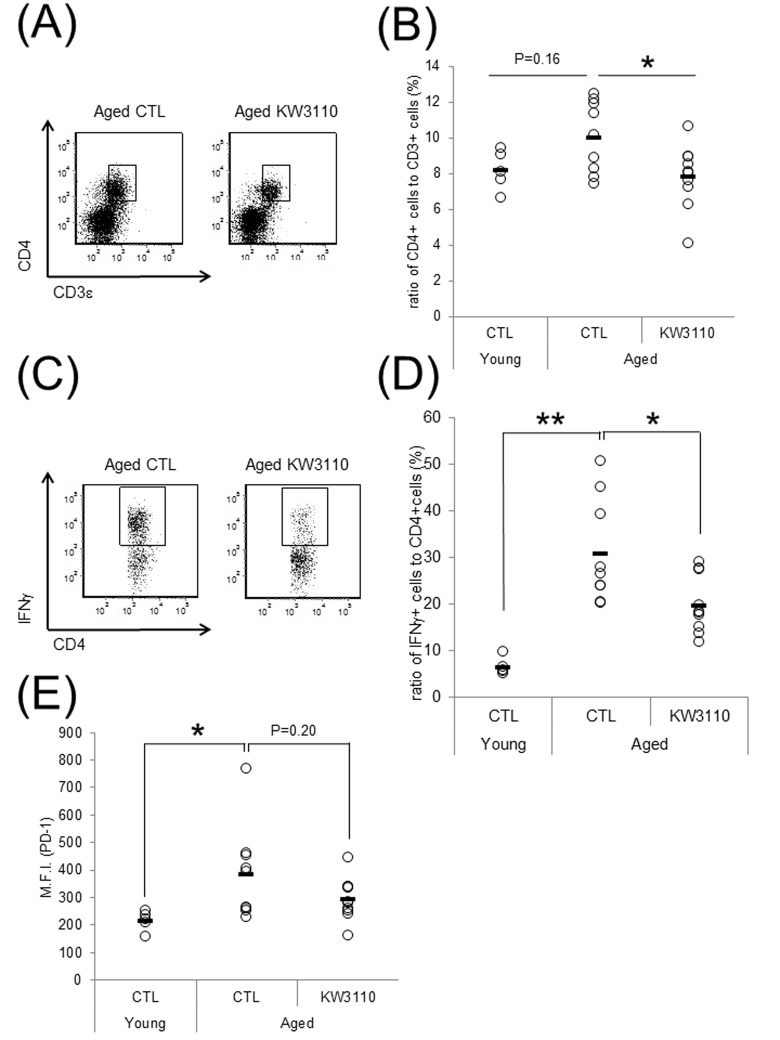
**Intake of *Lactobacillus paracasei* KW3110 suppressed the inflammatory CD4-positive T cell expansion in the lamina propia of the small intestine (SI-LP).** (**A** and **B**) To detect inflammatory cytokine-producing cells, SI-LP cells from young mice (3-months-old) and aged mice (17-months-old) were cultured under stimulation with Leukocyte Activation Cocktail plus BD GolgiPlug, and analyzed by flow cytometry. (**A**) Representative data of CD4-positive cells from aged mice fed a diet with (KW3110) or without (CTL) *L. paracasei* KW3110. (**B**) The ratio of CD3ε- and CD4-positive to live cells. (**C**) Representative data of CD4- and interferon gamma (IFN-γ)-positive cells from aged mice fed a diet with or without *L. paracasei* KW3110. (**D**) The ratio of CD4- and IFN-γ-positive cells to CD4-positive cells. (**E**) The expressions of programmed cell death protein 1 (PD-1) in CD3ε- and CD4-positive cells were analyzed by flow cytometry. M.F.I. indicates mean fluorescence intensity.

We have previously shown that orally-provided *L. paracasei* KW3110 interacted with immune cells in the small intestine [43]. In addition, intake of *L. paracasei* KW3110 altered gut bacterial flora composition in aged mice (Fig. 1). Thus, to examine the effects of *L. paracasei* KW3110 on the immune system in the small intestine with aging, 11-month-old mice were fed a diet with or without *L. paracasei* KW3110 for 6 months. The ratio of CD3ε- and CD4-double positive T cells to live cells and the ratio of interferon-γ (IFN-γ)-producing CD4-positive T cells to CD4-positive T cells, known as indicators of age-related inflammation in SI-LP cells, in aged mice fed a control diet, was higher than that in control young mice (Fig. 2B, D). The expression of programmed cell death protein 1 (PD-1), known as an indicator of immune senescence in CD4-positive T cells, in aged mice fed a control diet, was also higher than that in control young mice (Fig. 2E). However, the intake of *L. paracasei* KW3110 for 6 months in aged mice significantly decreased the ratio of CD3ε- and CD4-double-positive T cells to live cells in SI-LP (Fig. 2A, B), the ratio of IFN-γ-producing CD4-positive T cells to CD4-positive T cells (Fig. 2C, D), and the expression of PD-1 in CD4-positive T cells (Figure 2E). In contrast, the ratio of CD4- and Foxp3-positive cells, known as regulatory T cells, in SI-LP was not changed in aged mice fed a diet either with or without *L. paracasei* KW3110 (Supplementary Fig. 1).

### Intake of *L. paracasei* KW3110 decreased the levels of proinflammatory cytokines and chemokines in serum

**Figure 3:**
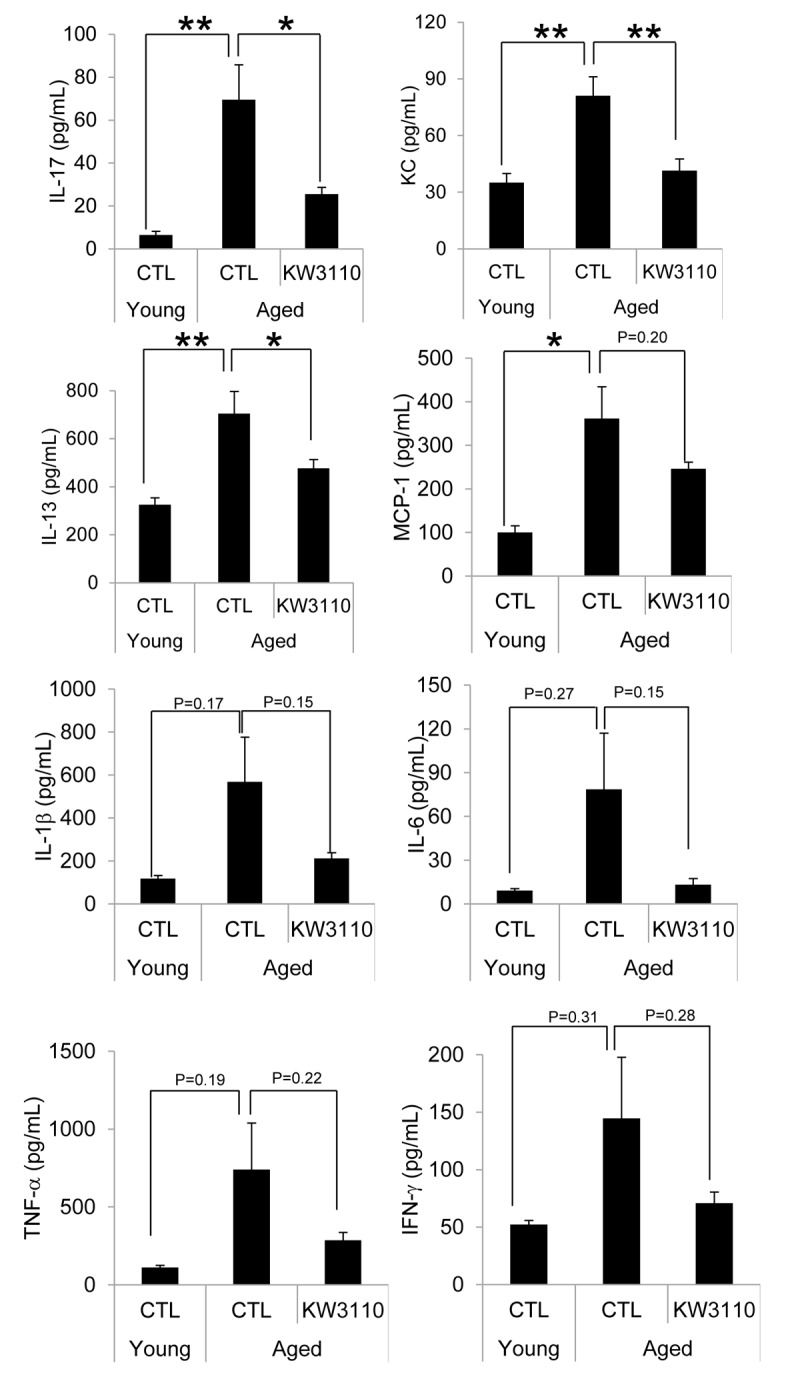
**The levels of proinflammatory cytokines in the serum were mitigated in aged mice fed a diet containing *Lactobacillus paracasei* KW3110 as compared with age-matched control mice.** Serum was collected and subjected to multiplex analyses to determine levels of cytokines (IL-1β, IL-6, IL-13, IL-17, IFN-γ, TNF-α, KC, and MCP1) in young (3-months-old) and aged mice (22-months-old). Values are presented as the means ± SEM. Significance was assumed if the p value was < 0.05. ^*^p < 0.05; ^**^p < 0.01. CTL = control diet; KW3110 = *Lactobacillus paracasei* KW3110 diet; IL = interleukin; IFN = interferon; TNF = tumor necrosis factor; KC = keratinocyte chemoattractant; MCP1 = monocyte chemoattractant protein 1.

The age-related inflammatory phenotypes in various tissues are associated with the serum levels of proinflammatory cytokines, which are produced from inflammatory immune cells. Therefore, we evaluated serum levels of proinflammatory cytokines and chemokines in aged mice. As shown in Fig. 3, the serum levels of proinflammatory cytokines and chemokines in aged mice were higher than those in control young mice (Fig. 3). Interestingly, serum levels of some cytokines and chemokines, interleukin-17 (IL-17), keratinocyte chemoattractant (KC), and interleukin-13 (IL-13), were significantly lower in aged mice fed a diet containing *L. paracasei* KW3110 from 16 months of age to 22 months of age for 6 months, than in age-matched mice fed a control diet (Fig. 3). The concentrations of the other proinflammatory cytokines were also lower in aged mice fed a diet containing *L. paracasei* KW3110 for 6 months. These changes of proinflammatory cytokine levels could be observed in aged mice fed a diet containing *L. paracasei* KW3110 only for 2 months (Supplementary Fig. 2).

### Intake of *L. paracasei* KW3110 mitigated retinal inflammation

**Figure 4:**
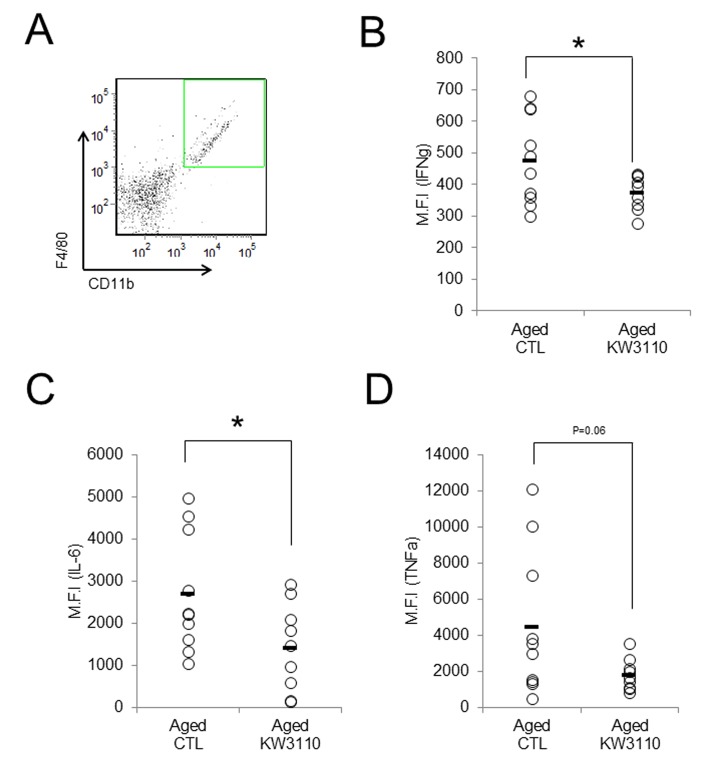
**Intake of *Lactobacillus paracasei* KW3110 mitigated retinal inflammation.** Intake of *L. paracasei* KW3110 in aged mice (17-months-old) suppressed the expression of inflammatory cytokines in retinal macrophage of aged mice. F4/80 and CD11b-positive macrophage in retina were gated as shown in (**A**), and median fluorescent intensity of intracellular IL-6, IFN-γ, and TNF-α were analyzed by flow cytometry (**B**-**D**). Significance was assumed if the p value was < 0.05; ^*^p<0.05. All abbreviations are defined in the Figure 3 legend.

As the lower serum levels of proinflammatory cytokines in aged mice were seemingly related to the suppression of age-related inflammatory phenotypes in peripheral tissues, we investigated whether intake of *L. paracasei* KW3110 also mitigated age-related retinal inflammation. Intake of *L. paracasei* KW3110 from 11–17 months of age for 6 months, in aged mice, significantly decreased the expression of IFN-γ and interleukin-6 (IL-6) in CD11b-positive and F4/80-positive retinal immune cells and macrophage (Fig. 4A-C) as compared with in age-matched mice fed a control diet. The expression of tumor necrosis factor-α (TNF-α) in CD11b-positive and F4/80-positive retinal macrophage was also lower than in age-matched mice fed a control diet (Fig. 4D).

### Intake of *L. paracasei* KW3110 suppressed age-related retinal cell loss

**Figure 5:**
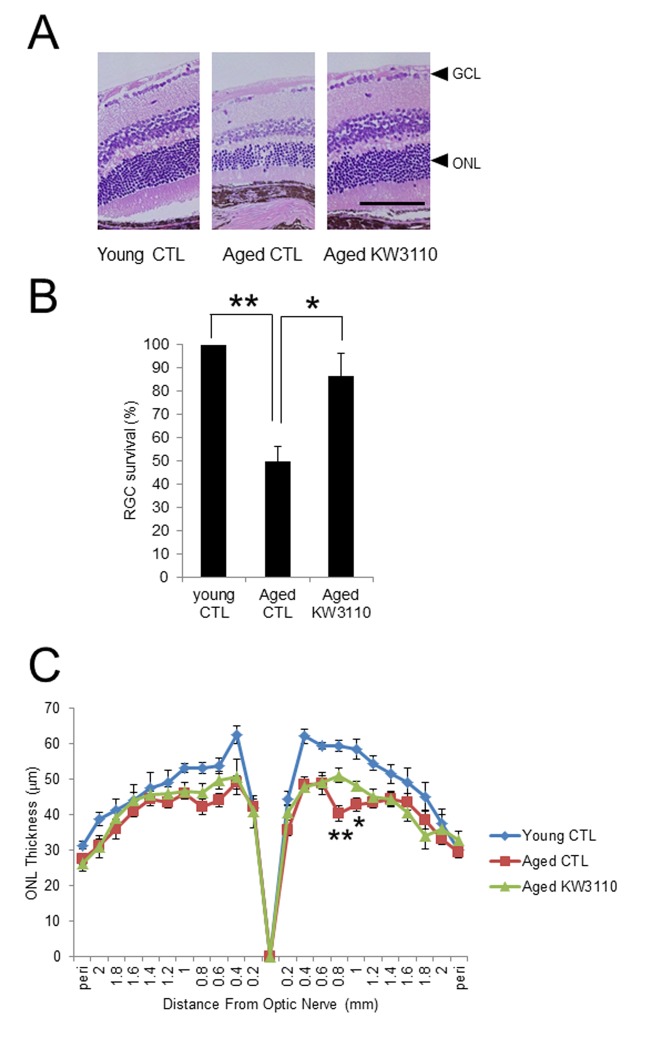
**Protective effect of *Lactobacillus paracasei* KW3110 on age-induced histological changes and ganglion cell loss in the retina.** (**A**) Hematoxylin and eosin staining of retinal sections in young mouse (3-months-old) fed a control (CTL) diet and aged mice (22-months-old) fed a diet either with or without *L. paracasei* KW3110 (KW3110 diet). Arrow heads indicate the ganglion cell layer (GCL) and outer nuclear layer (ONL), respectively. Scale bar represents 100 μm. (**B**) The survival rate of retinal ganglion cells (RGCs) in aged mice (22-months-old) fed a diet either with or without *L. paracasei* KW3110 (KW3110 diet) were analyzed compared to the survival of RGCs in young mice (3-months-old) fed a control diet. Values are presented as the means ± SEM. Significance was assumed if the p value was < 0.05. ^**^p < 0.01. (**C**) ONL thickness was lower in aged mice (22-months-old) fed control diet than in aged mice fed a diet with *L. paracasei* KW3110. Values are presented as the means ± SEM. Significance was assumed if the p value was < 0.05. ^*^p < 0.05; ^**^p < 0.01.

To elucidate the effects of the intake of *L. paracasei* KW3110 on age-related inflammatory phenotypes in retina, the number of RGCs was counted (Fig. 5A). The survival of RGCs was significantly decreased in aged mice as compared with that of young control mice. However, the survival of RGCs was significantly improved in aged mice fed a diet containing *L. paracasei* KW3110 from 16–22 months of age for 6 months (Fig. 5A, B), as compared with age-matched mice fed a control diet. In addition, the outer nuclear layer (ONL) thickness, corresponding to the photoreceptor layer, in aged mice fed a diet containing *L. paracasei* KW3110 for 6 months was also significantly thicker than in age-matched mice fed a control diet. These results were consistent with the suppressive results of proinflammatory cytokine levels in aged mice fed a diet containing *L. paracasei* KW3110.

## DISCUSSION

Defective immune functions with aging are key triggers of age-related chronic inflammatory diseases, including infectious diseases, tumors, diabetes, and neurodegenerative diseases [8]. With rapid increases in the aging population, the prevention of age-related immunological dysfunctions and chronic inflammation are necessary to extend the healthy lifespan. In the present study, we demonstrated that long-term intake of heat-killed *L. paracasei* KW3110 in aged mice significantly enhanced the population of beneficial gut bacteria, of the *Bifidobacterium* family*,* and slowed the age-related immune dysfunctions, expansion of the inflammatory IFN-γ-producing CD4-positive T cells in SI-LP, and lowered the serum levels of proinflammatory cytokines. We also found that intake of *L. paracasei* KW3110 mitigated retinal inflammation and age-related retinal cell death, probably associated with the prevention of age-related retinal diseases.

Age-related alterations of gut microbiota composition have been reported to cause immune senescence and intestinal chronic inflammation [34,47]. Although some probiotics improve the intestinal environment and suppress inflammation, the effects of long-term ingestion of probiotics remain unclear. In the present study, we showed that intake of *L. paracasei* KW3110 improved the age-related changes of the gut microbiota composition (Fig. 1). The *Firmicutes* to *Bacteroidetes* bacterial ratio increased in aged mice fed a control diet as compared with aged mice fed a diet containing *L. paracasei* KW3110 for 6 months. The age-related increase of *Firmicutes* to *Bacteroidetes* ratio was consistent with a previous study [48]. An increased *Firmicutes* to *Bacteroidetes* ratio has been reported to be associated with intestinal inflammation in obese patients [49]. The long-term intake of *L. paracasei* KW3110 might mitigate intestinal inflammation and energy metabolic disorders by modulating the *Firmicutes* to *Bacteroidetes* ratio. The intake of *L. paracasei* KW3110 also increased the relative abundance of *Bifidobacteriaceae* families (Fig. 1D). *Bifidobacterium* is known as one of the most beneficial bacterial family, though the bacteria is not detected in the elderly [44,50,51]. In addition, the intake of *Bifidobacterium* has been reported to result in decreased levels of proinflammatory cytokines, such as TNF-α, in the elderly [44]. In contrast, intake of *L. paracasei* KW3110 decreased the relative abundance of *Streptococcaceae* (Fig. 1D). In a previous report, the *Streptococcaceae* bacteria stimulated the intestinal cells to induce CCL20 chemokine production [52] and inflammatory IFN-γ-producing CD4-positive T cells were attracted by the CCL20 chemokine [53]. In this study, the intake of *L. paracasei* KW3110 for 6 months in aged mice also significantly reduced *Ccl20* gene expression in SI-LP as compared with that of age-matched control mice (Supplementary Fig. 3). These results suggested that the mitigation of age-related alterations in gut microbiota composition by the intake of *L. paracasei* KW3110 was important to suppress age-related intestinal chronic inflammation.

Indeed, the intake of *L. paracasei* KW3110 in aged mice significantly suppressed the age-related increase of inflammatory CD4-positive T cells, producing inflammatory cytokines (IFN-γ) at high levels in the SI-LP (Fig. 2). The expression of PD-1, one of the senescence markers, in CD4-positive T cells in SI-LP was also lower in aged mice fed a diet containing *L. paracasei* KW3110 than that of aged mice fed a control diet. These anti-inflammatory effects on intestinal immune cells might be due to mitigation of age-related decreases of *Bifidobacterium*. The modulatory effects on intestinal immune cell subpopulations might be associated with a direct interaction between *L. paracasei* KW3110 and intestinal immune cells. In a previous study, our group showed that *L. paracasei* KW3110 interacted with intestinal macrophages and suppressed excessive inflammation, including dermatitis, in mice and humans [40–42]. Because lactic acid bacteria, including *L. paracasei* KW3110, have some toll-like receptor (TLR) ligands, such as lipoteichoic acid, *L. paracasei* KW3110 might modulate intestinal immune cell activation by a TLR-dependent pathway. Further studies are required to determine the mechanism underlying the relationship between the intake of *L. paracasei* KW3110 and suppressive effects on age-related expansions of intestinal inflammatory immune cells.

Proinflammatory cytokines produced by intestinal inflammatory immune cells are possibly transferred to other tissues, including the retina, through the blood. Age-related visual function declines and eye diseases, such as AMD, might be associated with retinal inflammation, because retinal chronic inflammation is toxic to retinal cells, including photoreceptor cells and RGCs [16–19]. In the present study, the intake of *L. paracasei* KW3110 mitigated the inflammation in the retinal macrophage in aged mice (Fig. 4). In addition, the anti-inflammatory effects of *L. paracasei* KW3110 resulted in inhibition of age-related retinal cell death (Fig. 5). In previous studies, RGCs have been reported to mediate behaviors associated with response to light information and genetic ablation of RGCs results in loss of light-evoked behaviors [54–57]. We have obtained preliminary data that intake of *L. paracasei* KW3110 in aged mice could preserve the light-evoked locomotor activities as compared with age-matched control mice (data not shown). Although further studies, including immunohistochemical analysis, are needed, *L. paracasei* KW3110 might suppress age-related retinal cell death. Immunological phenomena are mainly regulated by macrophage in the retina. Macrophage consists of at least two subgroups, classic inflammatory M1 macrophage or alternative anti-inflammatory M2 macrophage [58,59]. M1 macrophages produce inflammatory cytokines, such as TNFα and IL-6, whereas M2 macrophages are considered to be associated with anti-inflammatory responses, including tissue remodeling, through the production of neurotrophic factors and anti-inflammatory cytokines [60,61]. Recently, we found that the intake of *L. paracasei* KW3110 suppressed light-induced retinal inflammation (unpublished data). Although further studies are required to evaluate the effects of *L. paracasei* KW3110 on macrophage activation in the retina, the inhibitory effects of *L. paracasei* KW3110 on age-related retinal cell death might be accompanied at least in part, by the regulation of macrophage activities. In addition, blood-borne macrophages have been reported to enter the retina via the optic nerve and ciliary body in light exposure mice model [62]. Our flow cytometry analysis showed that CD11b and f4/80-positive retinal cells, the retinal macrophages, in aged mice increased more than in young mice (Supplementary Fig. 4). Taken together, proinflammatory macrophages might penetrate into the retina under the age-related retinal degenerative condition.

In the present study, the anti-inflammatory effects of *L. paracasei* KW3110 on immune cells were observed in aged mice fed each diet from 11–17 months of age for 6 months (Figs. 2 and 4). However, in aged mice fed a diet containing *L. paracasei* KW3110 from 16–22 months of age for 6 months, such anti-inflammatory effects on immune cells were mild and not significantly different as compared with that of age-match control mice (data not shown). These results suggested that 22 months of age was too old to evaluate the effects of *L. paracasei* KW3110 on intestinal immune cells.

Lactic acid bacteria are considered to be phagocytosed by intestinal M cells. In a previous report, M cells in aged mice (18 months of age) were not fully functional [63]. This may be because the effects of *L. paracasei* KW3110 on immune cells in aged mice of 22 months of age were milder than in aged mice of 17 months of age. In the present study, we showed that intake of *L. paracasei* KW3110 from 16–22 months of age for 6 months significantly suppressed the serum levels of proinflammatory cytokines, alteration of gut microbiota composition, and retinal cell loss (Figs. 1, 3, and 5). Because the anti-inflammatory effects on serum cytokine levels were observed in aged mice fed a diet containing *L. paracasei* KW3110 (Supplementary Fig. 2), it was suggested that these phenotypes were reflected by the accumulation of anti-inflammatory effects for several months. In other words, continuous preventive methods, like dietary supplementation, might be much more effective in the delay of chronic inflammation.

In conclusion, the intake of *L. paracasei* KW3110 mitigated chronic inflammation in the intestine and retina, and reduced age-related retinal cell death. Further studies are needed to evaluate the effects in age-related senescent changes of the retina.

## MATERIALS AND METHODS

### Animals

The Mice (C57BL/6N, female) were purchased from Japan SLC (Hamamatsu, Japan). Young (1-month-old, n = 5), or aged (11-months-old, n = 10 in each group, or 16-months-old, n = 12 in each group) mice were acclimated until each aging study was started. Aged mice were divided by equal average weights into two groups. The control group mice were fed AIN93M (Oriental Yeast, Tokyo, Japan) and the *Lactobacillus paracasei* KW3110-fed mice (hereafter called the KW3110 group mice) were fed AIN93M containing 1 mg heat-killed *L. paracasei* KW3110/day/mouse for 6 months. The mice were housed in speciﬁc pathogen-free conditions under a 12-h light-dark photo cycle and had *ad libitum* access to water and the diet. The temperature in the room was kept at 25 ± 1°C and 60% ± 15% humidity.

All animal procedures and experiments were performed in accordance with the Association for Research in Vision and Ophthalmology Statement for the Use of Animals in Ophthalmic and Vision Research and institutional guidelines following approval by the Animal Care and Use Committee of National Center for Geriatrics and Gerontology (NCGG) (Obu, Japan).

### SI-LP and retinal cell preparations and flow cytometry analyses

The SI-LP cells and retinal cells were prepared for flow cytometric analyses. The SI-LP cells were prepared as described previously [64]. Retinas were digested with 1 mg/mL collagenase II (Worthington, Lakewood, NJ, USA) for 40 minutes at 37°C in Hanks' Balanced Salt Solution (HBSS) buffer with 1.0% bovine serum albumin (BSA). The tissue digest was then filtered through a 70 µm cell strainer, and washed with HBSS buffer with 1.0% BSA for 5 minutes at 1,300 rpm at 4°C. The supernatant was carefully removed and the digested tissue pellet was resuspended to form a single cell suspension. To investigate intracellular cytokine production, SI-LP and retinal cells were treated with a leukocyte activation cocktail with BD GolgiPlug (BD Biosciences, San Jose, CA, USA) for 4.5 hours and with a BD Cytofix/Cytoperm Fixation/Permeabilization kit (BD Biosciences), and then stained with the following antibodies to: CD3ε-BV421 (145-2C11) (Sony Biotechnology Inc., Tokyo, Japan), CD4-APC-Cy7 (GK1.5) (BioLegend, San Diego, CA, USA), IFN-γ-PE-Cy7 (XMG1.2) (eBiosciences, San Diego, CA, USA), TNF-α-BV421 (MP6-XT22) (BioLegend), IL-6-PE (MP5-20F3) (eBiosciences), IFN-γ-APC (XMG1.2) (eBiosciences), CD11b-APC-Cy7 (M1/70) (BD Biosciences), F4/80-PE-Cy7 (BM8) (BioLegend), programmed cell death protein 1 (PD-1)-BV421 (29F.1A12) (BioLegend), and 7-AAD (BD Pharmingen, San Jose, CA, USA). The 7-AAD− CD3ε- and CD4-positive cells were deﬁned as CD4-positive T cells and 7-AAD−CD11b-, and the f4/80-positive cells were defined as retinal macrophage cells. To detect Tregs, cells were treated with a Foxp3 Staining Kit (BD Biosciences) and stained with the following antibodies to: CD3ε-FITC (145-2C11) (eBiosciences), Foxp3-PE-Cy7 (FJK-16s) (eBiosciences), CD4-APC-Cy7 (GK1.5) (BioLegend), and 7-AAD (BD Pharmingen). The 7-AAD− CD3ε-, CD4-, and Foxp3-positive cells were defined as Tregs.

Data were collected using a FACS Canto II flow cytometer (BD Biosciences) and analyzed by FCS Express software (De Novo Software, Los Angeles, CA, USA).

### Analysis of serum cytokine concentrations

Blood samples were collected into heparin-coated tubes and centrifuged at 3,000 rpm for 5 min. Supernatants were collected and analyzed for serum cytokine concentrations using a Bio-Plex Pro mouse cytokine assay kit (Bio-Rad, Hercules, CA, USA).

### Analyses of gut microbiota

Feces were collected from young mice and aged mice fed control or KW3110-containing diets and stored at -80°C until further analyses. DNA was extracted according to a previous report [65]. Pyrosequencing of 16S ribosomal RNA was performed by Technosruga Lab (Shizuoka, Japan). The 16S ribosomal RNA sequencing was performed using the MiSeq^TM^II system (Illumina, San Diego, CA, USA) according to a previously described method [65]. The V3-V4 regions of 16S ribosomal RNA genes were amplified by PCR from fecal genomic DNA using the following universal primers [66,67]: 341F, 5′-AATGATACGGCGACCACCGAGATCTACACTCTTTCCCTACACGACGCTCTTCCGATCTCCTACGGGAGGCAGCAGCCTACGGGAGGCAGCAG-3′, and 806R, 5′-CAAGCAGAAGACGGCATAGAGATNNNNNNGTGACTGGAGTTCAGACGTGTGCTCTTCCGATCTGGACTACHVGGGTWTCTAAT-3′. PCR products were purified using a MultiScreen PCR filter plate (Merck Millipore, Darmstadt, Germany). All amplicons were sequenced using the paired-end, 2 × 250 bp cycle run on the MiSeq^TM^II system with a MiSeq Reagent Kit. The sequences were evaluated and filtered for their length and quality. Paired-end sequencing was joined using the fastq-join program (http://code.google.com/p/ea-utils/). The low quality reads, quality value scores < 20 for more than 99% of the sequence, were removed. The nucleotide sequence dataset was deposited in DNA Data Bank of Japan (DDBJ) with the accession numbers SAMD00118680-SAMD00118706.

Analyses of the extracted sequence reads were carried out using the Ribosomal Database Project (RDP) Multiclassifier version 2.11 [68] and Basic Local Alignment Search Tool (BLAST) search using the Metagenome@KIN analysis software (World Fusion, Tokyo, Japan). Reads showing > 97% similarity were grouped in each taxonomic rank.

### Measurements of outer nuclear layer thickness in the retina and retinal ganglion cell survival

Retinal sections, which included the optic nerve head to the most peripheral region of the retina, were fixed in neutral 10% formalin and stained with hematoxylin and eosin (HE), and the outer nuclear layer (ONL) thickness was measured in all areas and averaged at each point from the optic nerve head. The number of RGCs was counted within whole retinal sections.

### Statistical analysis

All values are expressed as the mean ± SEM. Statistical differences between three groups (young mice group fed a control diet, aged mice group fed a control diet, and aged mice group fed a diet containing KW3110) were analyzed by one-way analysis of variance (ANOVA), followed by the Tukey-Kramer test with signiﬁcance set at *p* < 0.05. Statistical differences between two groups (aged mice group fed a control diet versus aged mice group fed a diet containing KW3110) were determined using an unpaired, two-tailed Student's *t*-test with signiﬁcance set at *p* < 0.05. All statistical analyses were performed by using the Ekuseru-Toukei 2012 software program (Social Survey Research Information, Tokyo, Japan).

## Supplementary Material

Supplementary Figures
