# Supplementary material for: Long-term intake of Lactobacillus paracasei KW3110 prevents age-related chronic inflammation and retinal cell loss in physiologically aged mice
Source: Aging (Albany NY). 2018 Oct 19;10(10):2723–40. doi: 10.18632/aging.101583 (PMC6224250; doi:10.18632/aging.101583)
Supplement: Supplementary Figures [file aging-10-101583-s001.pdf]

SUPPLEMENTARY FIGURES

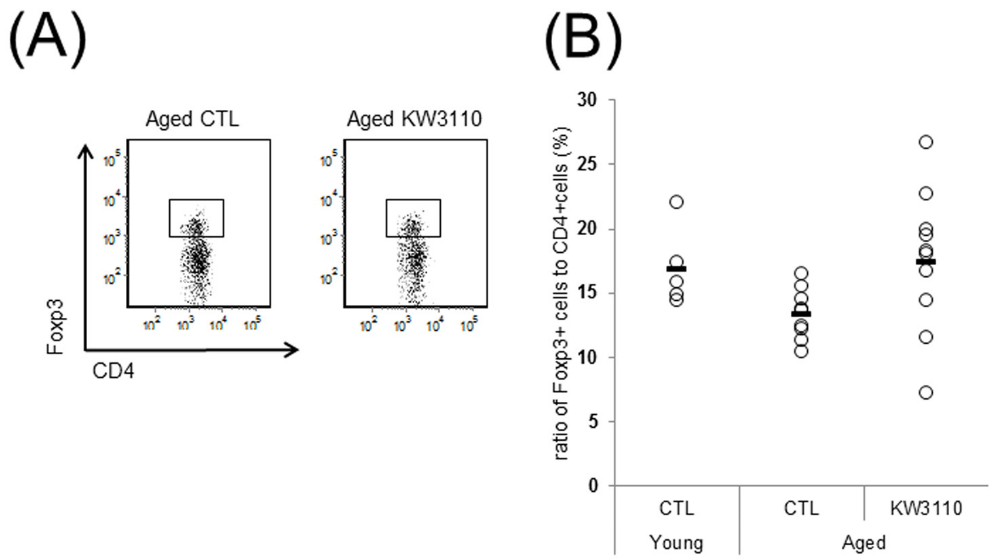

Supplementary Figure 1. Intake of *Lactobacillus paracasei* KW3110 did not affect the regulatory T cells (Tregs) population in the lamina propia of the small intestine (SI-LP). (**A** and **B**) To detect Tregs, SI-LP cells from young mice (3-months-old) and aged mice (17-months-old) were cultured and analyzed by flow cytometry. (**A**) Representative data of CD3e-, CD4-, and Foxp3-positive cells from aged mice fed a diet with or without *L. paracasei* KW3110. (**B**) The ratio of CD3ε-, CD4-, and Foxp3-positive to CD3ε- and CD4-positive cells. Statistical differences between three groups (young mice group fed a control diet, aged mice group fed a control diet, and aged mice group fed a diet containing KW3110) were analyzed by one-way analysis of variance (ANOVA), followed by the Tukey-Kramer test with significance set at $p < 0.05$. CTL = control diet; KW3110 = *Lactobacillus paracasei* KW3110 diet.

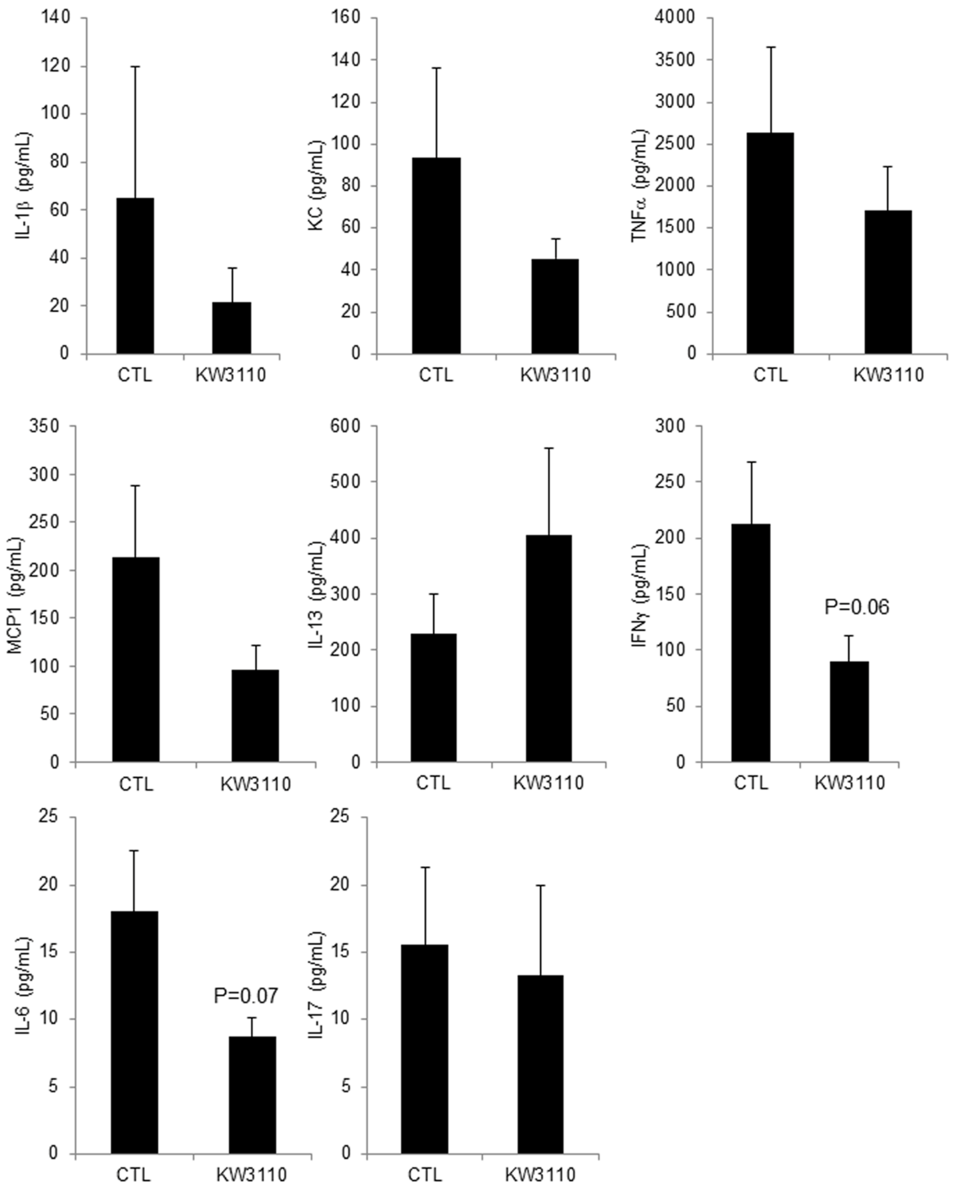

Supplementary Figure 2. The levels of cytokines in the serum of aged mice fed a diet either with or without *Lactobacillus paracasei* KW3110 for 2 months. Serum was collected and subjected to multiplex analyses to determine levels of cytokines (IL-1β, IL-6, IL-13, IL-17, IFN-γ, TNF-α, KC, and MCP1) in aged mice (18-months-old) fed a diet with (KW3110) or without (CTL) *L. paracasei* KW3110 for 2 months. Values are presented as the means ± SEM. Statistical differences between two groups (aged mice group fed a control diet versus aged mice group fed a diet containing KW3110) were determined using an unpaired, two-tailed Student's *t*-test with significance set at $p < 0.05$. IL = interleukin; IFN = interferon; TNF = tumor necrosis factor; KC = keratinocyte chemoattractant; MCP1 = monocyte chemoattractant protein 1.

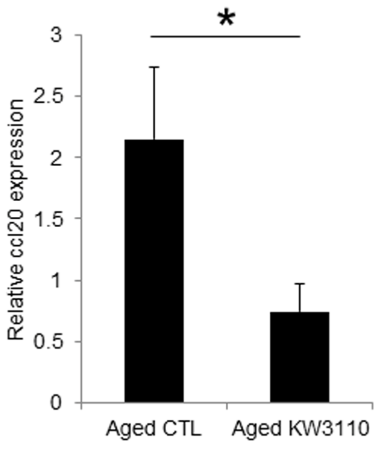

Supplementary Figure 3. Suppression of chemokine expression in the lamina propia of the small intestine (SI-LP) by intake of *Lactobacillus paracasei* KW3110. Total mRNA was extracted from SI-LP cells from aged mice (17-months-old) fed a diet with (KW3110) or without (CTL) *L. paracasei* KW3110 for 6 months. *Ccl20* gene expression was estimated by qRT-PCR and normalized to *Gapdh* gene expression. Values are presented as the means ± SEM. Significance was assumed if the p value was < 0.05. $^{*}p < 0.05$.

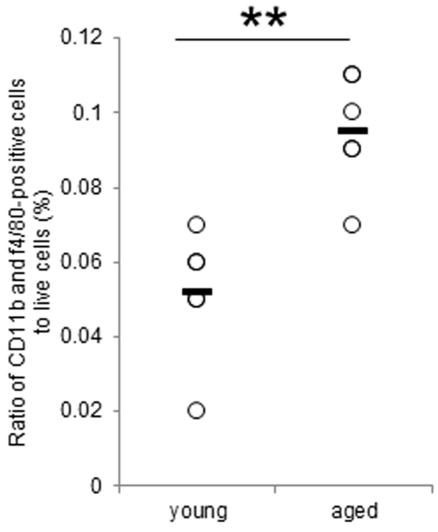

Supplementary Figure 4. The retinal macrophages increased in aged mice as compared with young mice. The ratio of CD11b and f4/80-positive to live cells. Significance was assumed if the p value was < 0.05; *p<0.05.
